# Intensive home treatment for adolescents in psychiatric crisis

**DOI:** 10.1186/s12888-019-2407-x

**Published:** 2019-12-19

**Authors:** Jet B. Muskens, Pierre C. M. Herpers, Caroline Hilderink, Patricia A. M. van Deurzen, Jan K. Buitelaar, Wouter G. Staal

**Affiliations:** 1 Karakter, Child and Adolescent Psychiatry, University Centre, Reinier Postlaan 12, 6525 GC Nijmegen, The Netherlands; 2 Department of Psychiatry, Radboud University Medical Centre, Donders Institute for Brain, Cognition and Behaviour, Kapittelweg 29, 6525 EN Nijmegen, The Netherlands; 3 Department of Cognitive Neuroscience, Donders Institute for Brain, Cognition and Behavior, Radboudumc, Nijmegen, The Netherlands; 4 Faculty of Social Sciences, Leiden Institute for Brain and Cognition, Postzone C2-5, P.O.Box 9600, 2300 RC Leiden, The Netherlands

**Keywords:** Adolescent psychiatry, Clinical admission, Intensive home treatment, Crisis intervention, High & intensive care

## Abstract

**Background:**

Adolescents with acute psychiatric disorders are typically treated with long-term clinical admission. However, long term admission may be associated with a variety of negative outcomes. This pilot study presents a new model of care, that is, the combined application of intensive home treatment and the possibility of short term stay at a psychiatric high & intensive care.

**Methods:**

In total 112 referred adolescents with mixed diagnoses participated in this longitudinal observational design. Clinical outcome was measured by the Health of the Nation Outcome Scales for Children and Adolescents (HoNOSCA) which measures the severity of multiple mental health problems. The HoNOSCA was clinician-rated at intake, after two months and after four months at discharge. Change in HoNOSCA total score was analysed with paired t-tests. Outcome moderators were gender, age, primary diagnosis, clinical admission, home treatment-time, medication and additional therapies. Follow up data were completed for 62 patients after two months and for 53 after four months.

**Results:**

Participants aged between 11 and 18 years (*M* = 14.8 years, SD = 0.3; 52% female). Mean HoNOSCA total score at intake was 18.8 (SD = 5.2), after two months 13.0 (SD = 5.0); after four months resulting in a score of 9.3 (SD = 5.2). None of the moderators tested showed a significant effect on HoNOSCA scores. However, a control group could not be used because of the severe psychopathology and high risk for suicidality and the lack of an effective treatment intervention for a comparable study group.

**Conclusion:**

With a symptom decrease of over 50% within four months as measured by the HoNOSCA, including less risk for hospitalization, this new model appears promising and of clinical relevance. Nevertheless, further research regarding stability of treatment outcome is warranted and evaluation of long-term effects of this model in follow-up studies is needed.

## Background

In the Netherlands, adolescents with severe psychiatric disorders typically used to be admitted to a psychiatric ward for several months [1]. However, long term psychiatric admission may be associated with a variety of negative outcomes, such as feeling displaced from home, family and friends, stagnation in social and emotional development or relapse of crisis because of lack of involvement of patients’ networks in regular treatment and is very expensive [2, 3].

Consequently, mental health care policy is now moving from long term admission towards intensive but short admission, followed by forms of intensive home treatment (IHT). Also, a clear need for more cost-effective forms of treatment is emerging. In search for more (cost-)effective treatment modalities, mental health services should be provided in the least restrictive setting, leading to different methods of outpatient treatment such as Crisis Resolution Teams implemented in the United Kingdom [4–6]. This was introduced in adult mental health care and research has shown outpatient treatment to reduce the number and duration of admissions, increase satisfaction of patients, decrease family burden and be more cost-effective [4–8].

In children and adolescents both intensive community services and inpatient care have been found to be associated with clinical improvements in most studies [9]. Thereby, intensive community services were associated with shorter hospitalizations, greater patient satisfaction and lower costs [9]. Home-based multisystem therapy (MST) showed to be effective at decreasing externalizing symptoms, improving family functioning and school attendance, together with higher satisfaction scores randomly assigned to inpatient hospitalization [10, 11]. Supported discharge services provided by an intensive community treatment team reduced psychiatric inpatient care for adolescents at 6 months follow-up compared to usual admission care, without differences in functional status and symptoms of mental health disorders between groups [12]. However, while developing new treatment models for youth in psychiatric crisis and moving from inpatient to outpatient treatment, inpatient treatment can be warranted in individual cases [6]. As such, short term studies show promising results, whereas results of long-term follow-up and independent replication of the results of intensive community treatment in youth are urgency [9, 13, 14].

In January 2015 a new Child and Youth Act was introduced in The Netherlands, that states that local municipalities are responsible for their youth policy, including mental health provisions [15]. Partly due to this change, there was a need to provide intensive nonclinical treatment for adolescents in psychiatric crisis, for example, severe depression, food refusal, disabling obsessive-compulsive disorder, often accompanied with school refusal. We developed an intervention based on Crisis Resolution and Home Treatment principles in which IHT is provided with a maximum of four months [16]. Mental health professionals visit patients at their home and treat their family together with the patient. Before start or during IHT, there is a possibility of short admission (with a maximum of 2 weeks) at a psychiatric high & intensive care (HIC) unit, together with their caregivers. The same mental health professionals of IHT are involved in the treatment of the patient and their caregivers during this short admission at the HIC. Short admission was thought to be feasible, since research showed most health gains to occur during the first weeks [16–18]. IHT principles are primarily based on solution-focused therapy [19] and attachment based family therapy [20] although individual interventions (e.g., medication, cognitive behaviour therapy) can be provided as well. IHT strongly focuses on improvement of the relationship between patient and caregivers, reintegration into school, work and hobby, reducing self-harming behaviour and increasing the adolescent’s motivation for therapy. This may prevent clinical admission, developing a dependency of hospital environment and being stigmatized as adolescent. Monthly evaluations of the patient and families take place at the hospital by both a child and adolescent psychiatrist and psychologist. According to the diagnosis and needs of the patient, pharmacotherapy and psychological therapies may be added.

Up till now, no studies regarding the clinical outcome of IHT in combination with the possibility of short admission of the adolescent with caregivers at a HIC in a child- and adolescent psychiatric setting have been published.

This study aimed to investigate treatment outcome of IHT, combined with HIC, by measuring the clinical outcome of adolescents with severe psychiatric crisis. As such, adolescents with a broad spectrum of psychiatric diagnoses, i.e. developmental disorders, eating disorders, anxiety and depression disorders, psychosis, comorbid disruptive behaviour disorders, personality disorders and symptoms of severe immediate risk to self and others were included in this study. Clinical functioning was established at start of treatment and after 2- and 4-months follow-up. Our hypothesis is that IHT will improve clinical outcome of the adolescents and families and lower risk for hospitalization.

## Method

### Participants

Participants were children and adolescents aged 11–18 years with severe psychiatric symptoms in need of acute and intensive treatment. Participants were required to have a minimum average estimated intelligence (IQ > 70). Estimated intelligence was based on either clinical functioning (e.g., good functioning at school) or by assessing an intelligence test (Wechsler Intelligence Scale for Children [21]). Data collection took place at Karakter Child and Adolescent Psychiatry in Nijmegen, The Netherlands. In 2014, the HIC was opened with a capacity to admit 6 patients. Adolescents with acute psychiatric disorder (age 11–18 year) can be admitted 24 h per day and 7 days per week. In case of voluntary admission in the HIC, presence of one of the caregivers during the first two days is required.

We collected data during treatment in which patients received IHT, with or without admission at the HIC for a maximum of two weeks. Inclusion was from 1/1/2015–28/2/2016. Patients who were admitted for over two weeks were excluded, as were patients younger than 11 years or older than 18 years of age. DSM-IV-TR [22] diagnoses were confirmed by a multidisciplinary team consisting of a child psychiatrist, a child psychologist and a nurse practitioner (developmental history, psychiatric and medical assessment of the child and a child and caregivers observation), and review of clinical and history records, including information from school and other professional institutions involved with the child [23]. Thus, a consensus diagnosis was allocated, which is seen as most reliable, compared to structured interviews when broad diagnostic categories are investigated [23, 24].

In the Netherlands, severe conduct problems and severe substance abuse are usually not treated within a psychiatric setting, but in juvenile welfare centres, juvenile penitentiary institutions or clinics for addiction problems. Hence, our clinic serves a specific population in which disruptive behaviour and substance abuse disorders are only seen as a comorbid disorder and not as primary diagnosis.

This study was designed as a longitudinal observational study and was approved by the Institutional Review Board of Karakter. The study does not fall under the Medical Research Involving Human Subjects Act, because there is no infringement of the physical and/or psychological integrity of the subject. For this reason, this study did not have to be reviewed by an accredited Medical Research Ethics Committee (MREC) or the Central Committee on Research Involving Human Subjects.

### Measure

The *Health of the Nation Outcome Scales for Children and Adolescents* (HoNOSCA) [25] was used to clinically assess the types and severity of mental health symptoms. The period of time that has to be rated comprises the previous two weeks. The HoNOSCA has shown to be a valid questionnaire, to require minimum time to be filled out and to have no physical or mental burden for subjects since it is filled out by their medical practitioners [26, 27]. The HoNOSCA consists of 15 scales and made up of two sections. The first section consists of 13 items relating to different types of problems; the second consists of two items relating the parent or young person’s knowledge of the nature of the young person’s difficulties and their information about the services available. A review article examining the psychometric properties of the HoNOSCA for children and adolescents indicates adequate construct and concurrent validity [28]. Since the inter-rater reliability of the second section (scales 14 and 15) are debated [25, 28, 29] we used a total score of scales 1–13, which makes a maximal total score of 52 points (higher score indicates more severe problems). Scales 1–13 are categorized in four categories, including behavioural problems (question 1–4), impairment (question 5–6), symptoms (question 7–9) and social problems (question 10–13). We divided scores by category, because focusing on individual items –rather than total scores– appears more useful in evaluating the impact of inpatient psychiatric treatment on adolescents [26]. Various studies have shown the HoNOSCA to be a feasible instrument in both in- and outpatient settings, and to have good inter-rater reliability and sensitivity to change at 3 months follow up [26, 27]. Furthermore, no effect on the HoNOSCA scores was found regarding the rater’s profession, experience or clinic, which makes it an excellent tool to use is multidisciplinary teams [30].

**Fig. 1:**
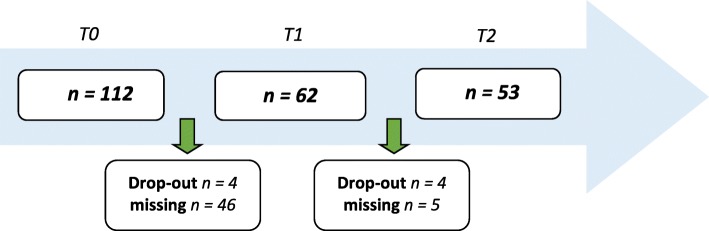
Available data at time of analyses. *T0* at intake, *T1* 2 months treatment, *T2* end of treatment period (4 months)

The HoNOSCA was filled out at 3 time points: at intake (T0); after 2 months of treatment (T1); after 4 months of treatment, that is, the end of IHT (T2). Scores were rated by trained mental health professionals from the IHT team (i.e., child and adolescent psychiatrists and psychologists). For the HoNOSCA assessed at T1, 46 were missing because assessment had not taken place. Of the remaining 62 at T1, two had one missing scale due to lack of patients’ cooperation to provide information. The data of T0, T1 and T2 was completed for 53 patients (Fig. 1).

### Data analysis

SPSS Statistics 22 was used for statistical analyses. Paired t-tests were run to compare data at T0 with T1, T1 with T2 and T0 with T2. Analyses were repeated with gender, age, primary diagnosis, admission and home treatment-time in minutes as moderators. Gender and admission were analysed running an independent sample t-test. Any correlation with age was tested using a Pearson Correlation test. To determine correlation with home treatment-time, a Pearson and Kendall’s tau test were used. The influence of the primary diagnosis was tested by running a univariate ANOVA model. To measure a change in HoNOSCA score of at least 4 points, which means complete improvement in one severe symptom or burden, we calculated the minimal needed number of subjects to be 27 (80% power, alpha = .05). To reduce the risk of type-1 error, we decided to use an alfa < 0.01 for our analysis of outcome variables.

## Results

### Descriptives

In total, data of 114 patients were available. Two patients were excluded (one was admitted involuntary; the other was 8 years of age). Of these 112 patients, 51.8% was female. All participants aged between 11 and 18 years (*M* = 14.8 years, SD = 0.3). There was no significant difference in age between boys (*M* = 14.6, SD = 0.4) and girls (*M* = 15.0, SD = 0.3). The sample was characterized by a range of psychiatric diagnoses by DSM-IV-TR, with autism spectrum disorder with emotion regulation problems (38%), anxiety disorders including post-traumatic stress disorders (20%) and depression disorders (19%) being most common (Table 1).

**Table 1:** Primary diagnosis

Primary diagnosis (*n* = 112)	Total	%	Female	%
Autism Spectrum Disorder	42	37.5	10	23.8
Depressive disorders	22	19.6	16	72.7
Anxiety disorders	21	18.8	13	61.9
Borderline personality disorder	9	8.0	8	88.9
Eating disorder	8	7.0	8	100
Disruptive disorders	3	2.7	0	0
Somatoform disorder	4	3.6	2	50
Psychosis	3	2.7	1	33.3
Intellectual disability (FSIQ < 85)	4	3.6	1	25

Fifteen patients (13%) were admitted during treatment for a maximum of two weeks. All admissions took place in between T0 and T1. The average duration of IHT treatment was 4–5 months with four patients treated for less than 3 months due to early dropping out and four patients treated for over 6 months due to admission by law preceding actual treatment and for precautionary safety reasons whilst waiting for follow-up treatment. Eight patients stopped treatment preliminary due to lack of motivation for therapy (two with an estimated FSIQ < 80, two with comorbidity of addiction disorder, two with eating disorder, two with disruptive behaviour disorder). These patients have been referred for other treatment options for these specific categories. Before referral, there was intensive discussion with patients, caregivers and professionals. In these cases, patients and caregivers did not feel that this intervention suited their needs as they expected. Hence they were not motivated to cooperate in the tasks they were given in therapy during IHT. These patients were referred to other health care services for treatment for these specific categories.

The data of T0, T1 and T2 was completed for fifty-three patients. By time of analyses, information about additional treatment was present for 65 patients: whom 12.3% received additional therapy such as cognitive behavioural therapy, 21.5% received pharmacotherapy and 26.3% received both additional therapy and pharmacotherapy.

By time of analyses, information about follow-up information was present for 60 patients of whom 45.0% remained in outpatient care within the same hospital, 38.3% received follow-up outpatient treatment elsewhere and 16.7% needed no further specialized treatment.

### HoNOSCA scores over time

**Fig. 2:**
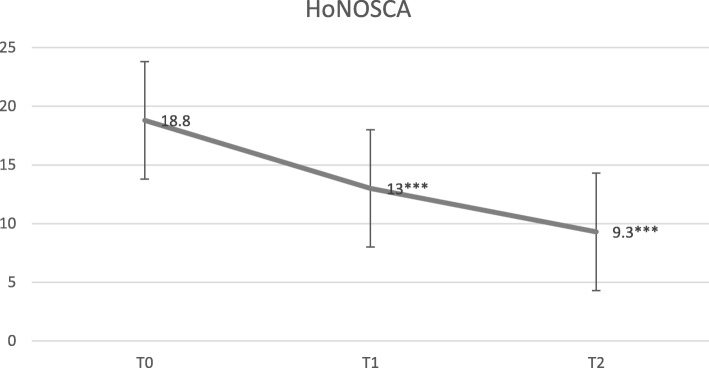
Mean HoNOSCA total scores. *T0* at intake, *T1* 2 months treatment, *T2* end of treatment period (4 months). *** *p* < .001

At T0, the mean HoNOSCA total scores of scales 1–13 was 18.82 (SD = 5.18). At T1, the mean HoNOSCA *total score* was 13.03 (SD = 5.00). At T2, the mean HoNOSCA total score was 9.40 (SD = 5.16). Table 2 and Fig. 2 show the mean HoNOSCA total scores by categories (*behaviour, impairment, symptoms* and *social*).

**Table 2:** HoNOSCA scores over time

	T0 (*n* = 112)			T1 (*n* = 62^a^)			T2 (*n* = 53)		
Section	Mean	SD	SEM	Mean	SD	SEM	Mean	SD	SEM
Behaviour	4.02	2.56	0.24	2.47	2.46	0.32	2.00	2.11	0.29
Impairment	1.00	0.15	1.56	0.42	0.88	0.11	0.34	0.76	0.10
Symptoms	5.00	2.39	0.23	3.21	2.14	0.27	2.06	2.17	0.30
Social	8.80	3.10	0.29	6.92	2.85	0.36	5.00	3.00	0.41
Total score^b^	18.82	5.18	0.49	13.03	5.00	0.65	9.40	5.16	0.71

^a^For section behaviour, *n* = 60

^b^HoNOSCA total score consists of scale 1–13

Table 3 shows the change in mean HoNOSCA total scores from T0 to T1, from T1 to T2 and from T0 to T2. All results show significant improvement with *p* < 0.01, except for the category *Impairment* from T1 to T2 (*p* = 0.164).

**Table 3:** Change in HoNOSCA score

	T0 - T1 (n = 62^a^)		T1 - T2 (n = 40) T0–T2 (n = 53)			
Section	Mean	SD	Mean	SD	Mean	SD
Behaviour	1.28^***^	2.24	0.90**	2.02	1.79^***^	2.50
Impairment	0.89^***^	1.69	0.18	0.78	0.98^***^	1.78
Symptoms	1.69^***^	1.97	1.18**	2.49	2.57^***^	2.57
Social	2.07^***^	3.30	1.95**	3.41	3.85^***^	3.90
Total score^b^	5.95^***^	6.44	4.20^***^	5.36	9.19^***^	6.92

*p < .05, ** *p* < .01, *** *p* < .001

^a^For section *Behaviour*, *n* = 60

^b^HoNOSCA total score consists of scale 1–13

### Moderators

We ran analyses with 7 variables, that is, gender, age, primary diagnosis, clinical admission yes/no, home treatment-time, additional therapy and medication, to determine whether these had a moderating effect on the height of mean HoNOSCA total scores at T0, T1 and T2. However, we found no statistically significant effects.

### Missing data

An independent samples t-test was run to define similarity between patients with data at T0, T1 and T2, compared to patients who had missing data at T1 and T2. No significant differences between responders and missing data were seen.

## Discussion

This study aimed to investigate treatment outcome of IHT, combined with HIC, by measuring the clinical outcome of 112 adolescents with severe psychiatric crisis. Clinical functioning was established at start of treatment and after 2- and 4-months follow-up. We found a statistically and clinically significant effect of IHT for adolescents in psychiatric crises, with a reduction of 53% on the mean HoNOSCA total scores after 4–5 months of treatment. Potential moderators, such as gender, age, primary diagnosis, clinical admission yes/no, home treatment-time, additional therapy and medication did not show significant effects. Despite a high dropout rate between intake and at discharge, no significant differences between responders and missing data were seen.

Our findings regarding clinical improvement in adolescents receiving IHT are consistent with other studies on the outcome of intensive community services in children and adolescents [9]. As such, our findings are also consistent with studies regarding cost-effectiveness. Previously, we have reported on cost-effectiveness, showing that the number of patients in treatment almost doubled while costs decreased substantially, with an overall reduction of the number of clinical admissions [1]. These findings are in line with studies that reported lower costs when providing intensive community services for children and adolescents [31]. As such, our findings are promising regarding positive treatment results, decreased need for clinical care and increased cost-effectiveness. Nevertheless, further research is needed to further verify our preliminary data.

In our study population, a minority of the patients used psychiatric medication. In general, antidepressants have shown to be efficacious [32]. However, there are limitations as well, such as an association with high nocebo effects (see review of Rojas-Mirquez et al. [33]), and findings that effects of (antidepressant) medication may be environment driven [34]. As such, it may not be surprising that psychiatric medication was not found to be a moderator for improvement on the HoNOSCA. However, it is important to further investigate the importance for psychiatric medication in either acute or chronic psychiatric disorders in adolescents. Moreover, it may be necessary to further investigate in which patient medication will be helpful. Since IHT focuses on increasing motivation for therapy, empowerment and improvement of the relationship between patient and parents, it seems to be important to investigate the relationship between psychological factors and the effect of medication as well.

This study has its limitations. First, study design and subsequently lack of a control group limit the validity of our findings. However, it is difficult to evaluate a comparable control group in a naturalistic setting, because of the severe psychopathology and high risk for suicidality in this study population. Thereby, at the time of this study, there were no other interventions available which specifically focused on replacement of hospitalization of this study population in the Netherlands. Moreover, the small group of patients that stayed over a longer period of time in our clinic, consisted of patients who were reluctant to enter therapeutic interventions and most of these patients were hospitalized involuntarily by Dutch law. Yet, as data on this topic are sparse, we believe the results of our pilot study to be of importance to the field. Furthermore, assessment was done by mental health professionals who were also patients’ clinicians. As such, rater-bias cannot be ruled out. Nevertheless, to retrieve reliable patient- and caretaker-ratings happened to be difficult due to low motivation to fill out these questionnaires. This raises questions regarding the therapeutic alliance, which has been found to predict symptom reduction [35]**.** Although we did not specifically study whether IHT showed any side effects, it could be speculated that discontinuation was related to treatment environment which did not fulfil the expectations of either patients or their caretakers. A further limitation is that we gathered data up to the end of IHT, whereas there remains a large need to further evaluate long term follow up, and to effective elements of these services. A last limitation to mention here is that this study was performed in a naturalistic setting, and for a large number of the patients who were referred to our acute psychiatric facilities, data were missing, which consequently lead to exclusion. Hence, we cannot rule out a positive selection bias.

## Conclusion

This pilot study focused on a patient group with severe psychopathology, for which previously long stay admission would have been necessary. The emotional and behavioural disturbances these patients show, do not only disrupt their personal lives, but the lives of their family members and friends as well. Treatment outcome of IHT was evaluated by measuring the clinical outcome by HoNOSCA at start of treatment and after 2- and 4-months follow-up. With a symptom decrease of over 50% within four months as measured by the HoNOSCA, this new model appears promising and of clinical relevance. As treatment of these children and adolescents is challenging and the search for more effective treatment interventions is going on, the findings seem to be important. Nevertheless, there remains a large need to further evaluate the outcome of intensive community services, and IHT specifically, especially with respect to long term follow up, and to effective elements of these services. Future studies should focus on long term follow-up of randomized controlled trials of different community based, outpatient care. Also, it would be interesting to evaluate the experience of the adolescents and the relationship between the adolescents, caregivers and mental health professionals in a qualitative design in future studies.

## Data Availability

The datasets generated and/or analyzed during the current study are not publicly available due to ethical restrictions and personal data protection. However, reasonable requests for patient level data should be made to the corresponding author and will be considered after discussion with the ethical board. As far as possible, we intend to include all relevant data in the manuscripts.

## Abbreviations

DSM-IV-TR: Diagnostic and Statistical Manual of Mental Disorders-4-Text Revision
FSIQ: Full-scale Intelligence Quotient
HIC: High & Intensive Care
HoNOSCA: Health of the Nation Outcome Scales for Children and Adolescents
IHT: Intensive Home Treatment
MREC: Medical Research Ethics Committee
MST: Multi System Therapy
